# Prevalence and influencing factors of workplace violence among psychiatric nurses in China: a cross-sectional study

**DOI:** 10.3389/fpubh.2026.1822574

**Published:** 2026-08-12

**Authors:** Liyun Deng, Zhou Wang, Guiying Cao, Xiangjun Li, Meng Zhou, Jiali Fan, Qiang Wang, Xiaojing Sun, Guangjian Li, Yun Yue, Jingping Shu

**Affiliations:** 1 Nursing Department, Changzhou Dean Hospital, Changzhou, Jiangsu, China; 2 Psychology Department, Changzhou Dean Hospital, Changzhou, Jiangsu, China; 3 Department of Psychosomatic Gastrointestinal Diseases, Changzhou First People’s Hospital, Changzhou, Jiangsu, China; 4 School of Humanities and Management, Wannan Medical University, Wuhu, Anhui, China; 5 Graduate School, Wannan Medical University, Wuhu, Anhui, China

**Keywords:** cross-sectional study, organizational support, perceived stress, psychiatric nurses, workplace violence

## Abstract

**Objective:**

To investigate the prevalence of workplace violence (WPV) among psychiatric nurses in China and to identify factors associated with WPV, thereby providing evidence for the prevention and management of workplace violence in psychiatric nursing practice.

**Methods:**

From September 2024 to September 2025, a cross-sectional survey was conducted among on-duty nurses from 20 psychiatric hospitals in Jiangsu Province, China. Data were collected using the Workplace Violence Scale (WVS), the Simplified Coping Style Questionnaire (SCSQ), the Chinese Perceived Stress Scale (CPSS), and the Survey of Perceived Organizational Support (SPOS). Multivariable logistic regression analyses were performed to examine factors associated with overall workplace violence, physical workplace violence, and non-physical workplace violence.

**Results:**

A total of 43.9% of psychiatric nurses reported experiencing at least one form of workplace violence during the past 12 months. Multivariable logistic regression analyses showed that sex, years of work experience, hospital level, department, number of monthly night shifts, and perceived organizational support were independently associated with overall workplace violence (all *p* < 0.05). Further analyses demonstrated that physical and non-physical workplace violence shared several common associated factors, including sex, years of work experience, hospital level, department, number of monthly night shifts, and perceived organizational support. Negative coping was independently associated only with non-physical workplace violence.

**Conclusion:**

Workplace violence remains highly prevalent among psychiatric nurses. Physical and non-physical workplace violence share common associated factors but may involve partly different underlying mechanisms. Physical workplace violence appeared to be more closely related to work-environmental and immediate patient-care conditions, whereas non-physical workplace violence was additionally associated with negative coping. These differences highlight the need to combine system-level prevention measures with targeted interventions addressing coping styles and non-physical aggression in psychiatric healthcare settings.

## 1 Introduction

Workplace violence (WPV) refers to any act, incident, or behavior that deviates from reasonable conduct and causes a person to be threatened, harmed, or physically injured in the course of work or as a direct consequence of work (1). It has been recognized by the International Labour Organization as a major occupational health problem worldwide. Previous studies have shown that WPV has become one of the most prominent occupational health challenges faced by healthcare workers globally. Medical staff are exposed to a relatively high risk of WPV during clinical practice, particularly in high-risk departments such as psychiatry and emergency medicine, where the incidence of WPV is significantly higher than in other healthcare settings (2–4).

Among healthcare professionals, nurses are widely regarded as a high-risk group for WPV due to the nature of their work and role characteristics, with high prevalence reported in both developed and developing countries (5). It has been estimated that approximately 25% of registered nurses have experienced physical assaults from patients or their relatives, while more than 50% have reported exposure to non-physical forms of WPV, such as verbal abuse or bullying (6). Frequent or prolonged exposure to workplace violence not only causes direct harm to nurses’ physical and mental health but also negatively affects job satisfaction, work engagement, and the quality of healthcare services, ultimately undermining the safe and effective functioning of healthcare systems (7, 8). Evidence further suggests that higher levels of workplace violence are associated with stronger turnover intention among nurses (9, 10).

Psychiatric nurses are considered a particularly high-exposure group to WPV due to the unique characteristics of their patient population and working environment (11). Psychiatric nursing care primarily involves patients with various mental disorders, some of whom may exhibit aggressive, hostile, or threatening behaviors as a result of disease-related symptoms, such as emotional dysregulation, impaired impulse control, cognitive dysfunction, or reduced reality testing capacity (12). During inpatient care, clinical assessment, medication administration, and behavioral interventions, psychiatric nurses are required to maintain frequent and close contact with patients. This intensive and sustained interpersonal interaction substantially increases their exposure to verbal abuse, threats, and even physical assaults in the workplace (13).

In addition, psychiatric nursing is often characterized by long-term and continuous care demands. Nurses commonly work in closed or semi-closed ward environments and must repeatedly manage symptom fluctuations, behavioral disturbances, and unexpected incidents, placing them under prolonged states of heightened vigilance and psychological tension (14). Compared with other clinical specialties, psychiatric nursing entails higher demands in emotional labor, risk prevention, and safety management, resulting in greater psychological burden and emotional exhaustion among nurses (15). Furthermore, occupational and organizational characteristics specific to psychiatric nursing may exacerbate work-related stress, weaken nurses’ psychological regulation and coping capacities, and may be associated with an increased likelihood of WPV (16). Therefore, psychiatric nurses are widely recognized as a high-risk population for workplace violence, warranting systematic investigation and sustained attention.

Existing studies have primarily examined WPV from the perspective of nurses in general hospitals or healthcare workers as a whole, whereas research focusing specifically on psychiatric nurses—a high-risk subgroup—remains relatively limited. Particularly in the Chinese context, there is a lack of large-scale, multicenter empirical evidence regarding the prevalence of WPV among psychiatric nurses, the characteristics of different types of violence and their associated factors. Previous literature suggests that psychosocial factors such as work-related stress, organizational support, and coping styles may be associated with workplace violence among nursing staff (17–19); however, their roles among psychiatric nurses have yet to be fully elucidated.

Within the Chinese psychiatric healthcare system, mental health services remain largely hospital-based, and substantial differences exist across regions and psychiatric hospitals in service capacity, workforce composition, and staffing resources (20). Shortages and unequal distribution of trained mental health professionals, together with the important role of nurses in mental health service delivery, may further increase the occupational demands placed on psychiatric nurses (21). In addition, workplace violence in China may be influenced by communication difficulties, mistrust between healthcare providers and service users, service-delivery processes, hospital security measures, and institutional mechanisms for reporting and responding to violent incidents and medical disputes (22). These systemic, organizational, and sociocultural factors may interact with ward-level demands and occupational role allocation, thereby shaping psychiatric nurses’ exposure to workplace violence and their capacity to prevent and manage violent incidents.

Given this background, a systematic investigation of WPV among psychiatric nurses based on a multicenter sample is necessary to enhance the representativeness and generalizability of the findings. Based on previous evidence, we hypothesized that demographic characteristics, occupational factors, perceived stress, coping styles, and perceived organizational support would be associated with workplace violence among psychiatric nurses. We further hypothesized that physical and non-physical workplace violence would share some associated factors while also exhibiting partly distinct patterns. Therefore, this study conducted a cross-sectional survey among nurses from 20 psychiatric hospitals in Jiangsu Province, China, to describe the prevalence of workplace violence experienced during the previous 12 months and to examine the demographic, occupational, and psychosocial factors associated with overall, physical, and non-physical workplace violence.

## 2 Methods

### 2.1 Study participants

From September 2024 to September 2025, registered nurses were recruited from 20 specialized psychiatric hospitals in Jiangsu Province, China. The participating hospitals included primary, secondary, tertiary, and tertiary grade A psychiatric hospitals and varied in size and service capacity. Eligible participants were registered nurses working in psychiatric or psychological inpatient or outpatient services at one of the participating psychiatric hospitals. The inclusion criteria were as follows: (1) holding a valid registered nurse qualification; (2) having at least 6 months of psychiatric nursing experience; (3) being actively engaged in psychiatric or psychological nursing services during the data-collection period; (4) aged 18 years or older; (5) able to understand and complete the questionnaire independently; and (6) voluntarily agreeing to participate and providing informed consent.

The exclusion criteria were as follows: (1) intern nurses; (2) visiting nurses undertaking short-term professional training; and (3) nurses who were on sick leave, maternity leave, or other extended leave during the data-collection period.

All questionnaire items were set as mandatory in the online survey system; therefore, there were no questionnaires with missing responses. Returned questionnaires were excluded from the final analysis if they contained obvious logical inconsistencies or were completed in less than 100 s, indicating insufficient engagement with the survey. This study was reviewed and approved by the Ethics Committee of Changzhou De’an Hospital (Ethics No. CZDALL-2024-006). All participants provided informed consent prior to participation.

### 2.2 Sample size calculation

The minimum sample size was estimated using the formula *n* = *Z*^2^_*α*/2_ *P*(1 *− p*)/*d*^2^, where n represents the minimum required sample size, *Z*_*α*/2_ represents the standard normal deviate corresponding to the confidence level, *p* represents the expected prevalence of workplace violence, and *d* represents the allowable absolute error. Previous studies have reported substantial variation in the prevalence of workplace violence among psychiatric nurses in China, ranging from 15% to 100% (23). Given this wide variation and to obtain the maximum required sample size, a conservative expected prevalence of 50% was used. With a 95% confidence level (*Z*_*α*/2_ = 1.96) and an allowable absolute error of 5% (*d* = 0.05), the minimum sample size was calculated as 384.16 and rounded up to 385. The adequacy of the sample size for the multivariable logistic regression analyses was additionally evaluated according to the number of outcome events per candidate predictor parameter. A total of 13 candidate predictors were considered. After accounting for the dummy coding of categorical variables, these predictors corresponded to a maximum of 20 regression parameters. Based on the conventional criterion of at least 10 outcome events per candidate predictor parameter (24), at least 200 outcome events were required for each model. The numbers of participants experiencing overall, physical, and non-physical workplace violence were 368, 279, and 332, respectively, corresponding to 18.4, 14.0, and 16.6 events per parameter. Therefore, the event counts were adequate for all three multivariable logistic regression models. Ultimately, 838 valid questionnaires were included in the analysis, exceeding the minimum sample size estimated using the prevalence-based formula and providing an adequate number of outcome events for the regression analyses.

### 2.3 Survey instruments

#### 2.3.1 General information questionnaire

A self-designed general information questionnaire was used to collect participants’ socio-demographic and work-related characteristics, including sex (male or female), age (years), marital status (unmarried, married, or divorced), years of work experience (<5, 5–10, 11–15, or >15 years), educational level (secondary specialized school, junior college, or bachelor’s degree or above), professional title (junior, intermediate, or senior), hospital level (primary, secondary, tertiary, or tertiary grade A hospital), department (outpatient department or inpatient ward), and the average number of night shifts per month (days). Age and the average number of night shifts per month were recorded as continuous variables, whereas the remaining variables were collected as categorical variables.

#### 2.3.2 Workplace violence scale (WVS)

The Workplace Violence Scale (WVS) was originally developed by Schat et al. (25) and subsequently translated and culturally adapted by Wang Peixi (26) to fit the Chinese cultural context and medical work environment, forming the Chinese version of the WVS for healthcare workers. This scale was used to assess the frequency of different types of workplace violence experienced by participants over the past year.

The scale consists of five dimensions: physical assault, emotional abuse, threats, verbal sexual harassment, and physical sexual harassment. Each item is rated on a 4-point scale (0–3), indicating 0 times, 1 time, 2–3 times, and ≥4 times, respectively. Higher scores indicate a higher frequency of exposure to the corresponding type of workplace violence in the past year.

In this study, physical assault and physical sexual harassment were categorized as physical violence, while emotional abuse, threats, and verbal sexual harassment were classified as non-physical violence. The scale has been widely used among healthcare workers, nurses, and psychiatric nurses, demonstrating good reliability and validity (27), and is suitable for assessing workplace violence in healthcare settings. In this study, the Cronbach’s *α* coefficient was 0.845, indicating good internal consistency.

#### 2.3.3 Simplified coping style questionnaire (SCSQ)

The Simplified Coping Style Questionnaire (SCSQ) was developed by Xie Yaning (28) based on both domestic and international coping theories and adapted to the characteristics of the Chinese population. The questionnaire consists of 20 items and includes two dimensions: positive coping (12 items, items 1–12) and negative coping (8 items, items 13–20). Each item is rated on a 4-point Likert scale ranging from 0 (“never use”) to 3 (“often use”). The positive coping dimension has a possible score ranging from 0 to 36, whereas the negative coping dimension ranges from 0 to 24. Higher scores on each dimension indicate a greater tendency to adopt the corresponding coping strategy. The SCSQ has been widely used to assess coping styles among Chinese healthcare workers and has demonstrated good reliability and validity (29). In the present study, the overall Cronbach’s *α* coefficient was 0.922.

#### 2.3.4 Chinese perceived stress scale (CPSS)

The Chinese Perceived Stress Scale (CPSS) was revised by Yang Tingzhong (30) based on the Perceived Stress Scale (PSS) developed by Sheldon Cohen et al. (31). It is designed to comprehensively assess individuals’ perceived stress during the previous month.

The CPSS consists of 14 items across two dimensions: perceived tension and perceived loss of control. Each item is rated on a 5-point Likert scale ranging from 0 (“never”) to 4 (“very often”). The total score ranges from 0 to 56, with higher scores indicating higher levels of perceived psychological stress. The CPSS has been extensively used in Chinese populations, including healthcare workers, and has demonstrated satisfactory reliability and validity (32). In the present study, the Cronbach’s *α* coefficient was 0.746.

#### 2.3.5 Survey of perceived organizational support (SPOS)

Perceived organizational support was assessed using the Survey of Perceived Organizational Support (SPOS) revised by Pan Xiaohua (33). This scale was adapted to the occupational characteristics of healthcare workers and consists of 14 items divided into two dimensions: emotional support (items 1–8) and instrumental support (items 9–14).

Each item is rated on a 5-point Likert scale ranging from 1 (“strongly disagree”) to 5 (“strongly agree”). The total score ranges from 14 to 70, with higher scores indicating higher perceived organizational support. The SPOS has demonstrated good reliability and validity among healthcare workers (34). In the present study, Cronbach’s *α* was 0.984.

### 2.4 Data collection

Data were collected using the online survey platform Questionnaire Star. The survey link was distributed to the nursing departments of the 20 participating psychiatric hospitals, which subsequently disseminated the questionnaire to eligible psychiatric nurses. Participation was voluntary and anonymous. Because the survey adopted an open invitation approach, the total number of nurses who received the invitation could not be accurately determined.

A total of 850 questionnaires were returned. Questionnaires completed in less than 100 s were excluded as part of the quality control process. Finally, 838 valid questionnaires were included in the analysis, yielding a valid questionnaire rate of 98.6%.

### 2.5 Statistical analysis

Statistical analyses were performed using SPSS version 26.0. The distributions of continuous variables were assessed using histograms, normal Q–Q plots, and skewness and kurtosis statistics. Overall, the continuous variables showed only mild deviations from normality and were considered approximately normally distributed. Continuous variables were expressed as means and standard deviations (SDs), and comparisons between groups were conducted using independent-samples t tests. Categorical variables were presented as frequencies and percentages, and group comparisons were performed using the chi-square test.

Potential common method variance was assessed using Harman’s single-factor test. All self-reported measurement items were entered into an unrotated principal component analysis. Common method variance was considered potentially substantial if the first unrotated factor accounted for more than 40% of the total variance.

Separate multivariable logistic regression analyses were performed to identify factors associated with overall workplace violence (WPV), physical workplace violence, and non-physical workplace violence. Based on theoretical considerations and previous literature, sociodemographic characteristics, work-related variables, perceived organizational support, perceived stress, and coping styles were included in the regression models. The same set of covariates was included in all multivariable logistic regression models to facilitate comparisons across the different types of workplace violence. Odds ratios (ORs) with 95% confidence intervals (CIs) were calculated. To assess the robustness of the estimated associations between sex and workplace violence given the unequal numbers of male and female participants, bootstrap sensitivity analyses with 1,000 resamples were conducted for each multivariable logistic regression model. Bias-corrected and accelerated (BCa) 95% confidence intervals were calculated for the sex coefficient. A two-sided *p* value < 0.05 was considered statistically significant.

## 3 Results

### 3.1 Common method variance test

Harman’s single-factor test yielded seven factors with eigenvalues greater than 1. The first unrotated factor explained 32.461% of the total variance, which was below the threshold of 40%, indicating that no substantial common method variance was identified.

### 3.2 General characteristics of participants and univariate analysis of workplace violence

A total of 838 psychiatric nurses were included in this study, including 101 males (12.1%) and 737 females (87.9%), with a mean age of 33.8 ± 7.7 years. Most participants were married (70.9%) and had a bachelor’s degree or above (82.2%). Regarding years of work experience, 17.2% had worked for <5 years, 31.7% for 5–10 years, 21.6% for 11–15 years, and 29.5% for >15 years. More than half of the participants worked in tertiary grade A psychiatric hospitals (56.1%), and the majority worked in inpatient wards (89.3%). Detailed demographic and work-related characteristics are presented in Table 1.

**Table 1:** Univariate analysis of workplace violence and associated factors among psychiatric nurses during the past 12 months (*n* = 838).

Variable	Categorization	WPV group (*n* = 368)	Non-WPV group (*n* = 470)	*χ*^2^/*t*	*p*
Sex	Male	59	42	9.806	0.002
	Female	309	428		
Age (years)		34.08 ± 7.18	33.60 ± 8.18	0.917	0.359
Marital status	Unmarried	87	141	4.906	0.086
	Married	272	322		
	Divorced	9	7		
Years of work experience	<5 years	42	102	29.204	<0.001
	5–10 years	122	144		
	11–15 years	105	76		
	>15 years	99	148		
Educational level	Secondary specialized school	4	4	2.830	0.243
	Junior college	53	88		
	Bachelor’s degree or above	311	378		
Professional title	Junior	167	249	6.177	0.046
	Intermediate	169	176		
	Senior	32	45		
Hospital level	Primary hospital	3	3	2.228	0.526
	Secondary hospital	77	94		
	Tertiary hospital	75	116		
	Tertiary grade A hospital	213	257		
Night shifts (days per month)		4.52 ± 2.54	3.75 ± 2.66	−4.225	<0.001
Department	Outpatient department	30	60	4.583	0.032
	Inpatient ward	338	410		
CPSS score	/	39.07 ± 7.09	36.75 ± 8.12	−4.392	<0.001
SPOS score	/	47.91 ± 12.65	54.25 ± 13.41	6.961	<0.001
Positive coping score	/	34.09 ± 7.68	37.17 ± 8.06	5.585	<0.001
Negative coping score	/	19.20 ± 4.83	18.94 ± 5.48	−0.706	0.480

WPV, workplace violence; CPSS, perceived stress score; SPOS, perceived organizational support score.

Among the 838 psychiatric nurses, 368 (43.9%) reported experiencing at least one workplace violence incident during the previous 12 months. Univariate analyses showed significant differences in workplace violence according to sex, years of work experience, professional title, department, number of monthly night shifts, perceived stress (CPSS), perceived organizational support (SPOS), and positive coping scores (all *p* < 0.05). Specifically, workplace violence was more common among male nurses, nurses working in inpatient wards, and those with more monthly night shifts. In addition, nurses in the workplace violence group had significantly higher CPSS scores but lower SPOS and positive coping scores than those in the non-workplace violence group. No significant differences were observed for age, marital status, educational level, hospital level, or negative coping scores (all *p* > 0.05). Detailed results are presented in Table 1.

### 3.3 Frequency distribution of different types of workplace violence

Among nurses who reported experiencing workplace violence during the previous 12 months, verbal abuse was the most frequently reported subtype (*n* = 313), followed by physical assault (*n* = 270) and verbal threats (*n* = 226). Verbal sexual harassment (*n* = 104) and physical sexual harassment (*n* = 76) were reported less frequently. Regarding frequency distribution, all types of workplace violence were most commonly reported as occurring once. The proportions of one-time occurrence were 42.49% for verbal abuse, 51.33% for verbal threats, 62.50% for verbal sexual harassment, 69.74% for physical sexual harassment, and 51.48% for physical assault. Notably, a proportion of nurses reported repeated exposure to workplace violence within the past year. Specifically, 27.80% of nurses experienced verbal abuse four or more times, and 16.30% experienced physical assault four or more times, indicating recurrent exposure to workplace violence among some psychiatric nurses during the past 12 months. Detailed results are shown in Table 2.

**Table 2:** Frequency distribution of different types of workplace violence among psychiatric nurses during the past 12 months (*n* = 838).

Frenquency	Verbal abuse (*n* = 313)		Verbal threats (*n* = 226)		Verbal sexual harassment (*n* = 104)		Physical sexual harassment (*n* = 76)		Physical assault (*n* = 270)	
	*n*	%	*n*	%	*n*	%	*n*	%	*n*	%
1 time	133	42.49	116	51.33	65	62.50	53	69.74	139	51.48
2–3 times	93	29.71	58	25.67	26	25.00	16	21.05	87	32.22
≥4 times	87	27.80	52	23.00	13	12.50	7	9.21	44	16.30

### 3.4 Multivariable logistic regression analysis of overall workplace violence

Using exposure to workplace violence (yes/no) as the dependent variable, a multivariable logistic regression model was constructed after appropriate coding of all prespecified covariates (Supplementary Table S1). Sex, years of work experience, hospital level, department, night shift frequency, and perceived organizational support were independently associated with workplace violence among psychiatric nurses (Table 3).

**Table 3:** Multivariable logistic regression analysis of factors associated with workplace violence among psychiatric nurses.

Factor	B	SE	Wald *χ*^2^	*p*	OR	95% CI
Sex (reference: female)	0.664	0.243	7.487	0.006	1.942	1.207 ~ 3.124
Age	0.011	0.025	0.217	0.641	1.012	0.964 ~ 1.062
Marital status (reference: divorced)			0.026	0.987		
Unmarried	−0.062	0.607	0.011	0.918	0.940	0.286 ~ 3.089
Married	−0.027	0.567	0.002	0.963	0.974	0.320 ~ 2.960
Years of work experience (reference: <5 years)			16.905	0.001		
5–10 years	0.827	0.311	7.060	0.008	2.287	1.242 ~ 4.211
11–15 years	1.203	0.404	8.867	0.003	3.330	1.509 ~ 7.352
>15 years	0.489	0.514	0.906	0.341	1.631	0.596 ~ 4.463
Educational level (reference: bachelor’s degree or above)			2.939	0.230		
secondary specialized school	0.878	0.816	1.157	0.282	2.405	0.486 ~ 11.904
Junior college	0.367	0.254	2.081	0.149	1.443	0.877 ~ 2.376
Professional title(reference: senior)			2.079	0.354		
Junior	−0.538	0.386	1.945	0.163	0.584	0.274 ~ 1.244
Intermediate	−0.275	0.307	0.803	0.370	0.759	0.416 ~ 1.386
Hospital level (reference: tertiary grade A)			11.006	0.012		
Primary	0.121	0.878	0.019	0.890	1.129	0.202 ~ 6.314
Secondary	−0.468	0.206	5.182	0.023	0.626	0.418 ~ 0.937
Tertiary	−0.572	0.193	8.817	0.003	0.565	0.387 ~ 0.823
Department (reference: outpatient department)	0.580	0.259	5.004	0.025	1.786	1.074 ~ 2.967
Night shifts (days per month)	0.112	0.034	10.974	0.001	1.119	1.047 ~ 1.196
SPOS score	−0.033	0.007	19.529	<0.001	0.968	0.954 ~ 0.982
CPSS score	0.015	0.011	1.710	0.191	1.015	0.993 ~ 1.038
Positive coping score	−0.017	0.013	1.607	0.205	0.983	0.958 ~ 1.009
Negative coping score	0.032	0.017	3.483	0.062	1.033	0.998 ~ 1.068
Constant	−0.710	1.348	0.278	0.598	0.492	

CI, confidence interval; CPSS, perceived stress score; OR, odds ratio; SPOS, perceived organizational support score.

Specifically, male nurses had significantly higher odds of experiencing workplace violence than female nurses (OR = 1.942, 95% CI: 1.207–3.124, *p* = 0.006). Compared with nurses with less than 5 years of work experience, those with 5–10 years (OR = 2.287, 95% CI: 1.242–4.211, *p* = 0.008) and 11–15 years of work experience (OR = 3.330, 95% CI: 1.509–7.352, *p* = 0.003) had significantly higher odds of workplace violence. Compared with nurses working in tertiary grade A psychiatric hospitals, those working in secondary (OR = 0.626, 95% CI: 0.418–0.937, *p* = 0.023) and tertiary psychiatric hospitals (OR = 0.565, 95% CI: 0.387–0.823, p = 0.003) had significantly lower odds of workplace violence. In addition, nurses working in inpatient wards had significantly higher odds of workplace violence than those working in outpatient departments (OR = 1.786, 95% CI: 1.074–2.967, *p* = 0.025). A higher number of monthly night shifts was associated with increased odds of workplace violence (OR = 1.119, 95% CI: 1.047–1.196, *p* = 0.001), whereas higher perceived organizational support was associated with lower odds of workplace violence (OR = 0.968, 95% CI: 0.954–0.982, *p* < 0.001). Detailed results are presented in Table 3.

### 3.5 Univariate analysis of physical workplace violence among psychiatric nurses

Univariate analyses showed significant differences in physical workplace violence according to sex, years of work experience, department, number of monthly night shifts, perceived stress (CPSS), perceived organizational support (SPOS), and positive coping scores (all *p* < 0.05). No significant differences were observed for age, marital status, educational level, professional title, hospital level, or negative coping scores (all *p* > 0.05). Detailed results are presented in Supplementary Table S2.

### 3.6 Multivariable logistic regression analysis of physical workplace violence among psychiatric nurses

Using exposure to physical workplace violence (yes/no) as the dependent variable, a multivariable logistic regression model was constructed after appropriate coding of all prespecified covariates (Supplementary Table S1). The results showed that sex, years of work experience, hospital level, department, number of monthly night shifts, and perceived organizational support were independently associated with physical workplace violence among psychiatric nurses (Table 4).

**Table 4:** Multivariable logistic regression analysis of factors associated with physical workplace violence among psychiatric nurses.

Factor	B	SE	Wald *χ*^2^	*p*	OR	95% CI
Sex (reference: female)	0.767	0.252	9.235	0.002	2.154	1.313 ~ 3.533
Age	0.019	0.028	0.472	0.492	1.019	0.965 ~ 1.077
Marital status (reference: divorced)			0.079	0.961		
Unmarried	−0.194	0.691	0.078	0.779	0.824	0.213 ~ 3.193
Married	−0.165	0.654	0.064	0.801	0.848	0.235 ~ 3.057
Years of work experience (reference: <5 years)			18.775	<0.001		
5–10 years	0.840	0.333	6.358	0.012	2.316	1.206 ~ 4.450
11–15 years	1.072	0.436	6.045	0.014	2.921	1.243 ~ 6.864
>15 years	0.066	0.566	0.014	0.907	1.068	0.352 ~ 3.242
Educational level (reference: bachelor’s degree or above)			3.637	0.162		
secondary specialized school	1.333	0.884	2.274	0.132	3.792	0.671 ~ 21.440
Junior college	0.364	0.276	1.734	0.188	1.439	0.837 ~ 2.472
Professional title(reference: senior)			1.906	0.386		
Junior	−0.580	0.448	1.677	0.195	0.560	0.233 ~ 1.347
Intermediate	−0.296	0.367	0.650	0.420	0.744	0.362 ~ 1.527
Hospital level (reference: tertiary grade A)			12.328	0.006		
Primary	−0.130	0.988	0.017	0.895	0.878	0.127 ~ 6.089
Secondary	−0.423	0.224	3.575	0.059	0.655	0.422 ~ 1.016
Tertiary	−0.736	0.217	11.491	0.001	0.479	0.313 ~ 0.733
Department(reference: outpatient)	0.884	0.324	7.433	0.006	2.422	1.282 ~ 4.573
Night shifts (days per month)	0.152	0.038	16.011	<0.001	1.164	1.081 ~ 1.254
SPOS score	−0.033	0.008	16.195	<0.001	0.968	0.953 ~ 0.983
CPSS score	0.019	0.013	2.331	0.127	1.020	0.955 ~ 1.045
Positive coping score	−0.010	0.015	0.493	0.483	0.990	0.961 ~ 1.019
Negative coping score	0.035	0.019	3.426	0.064	1.035	0.998 ~ 1.074
Constant	−1.860	1.516	1.505	0.220	0.156	

CI, confidence interval; CPSS, perceived stress score; OR, odds ratio; SPOS, perceived organizational support score.

Specifically, male nurses had significantly higher odds of experiencing physical workplace violence than female nurses (OR = 2.154, 95% CI: 1.313–3.533, *p* = 0.002). Compared with nurses with less than 5 years of work experience, those with 5–10 years (OR = 2.316, 95% CI: 1.206–4.450, *p* = 0.012) and 11–15 years of work experience (OR = 2.921, 95% CI: 1.243–6.864, *p* = 0.014) had significantly higher odds of physical workplace violence. Compared with nurses working in tertiary grade A psychiatric hospitals, those working in tertiary psychiatric hospitals had significantly lower odds of physical workplace violence (OR = 0.479, 95% CI: 0.313–0.733, *p* = 0.001). In addition, nurses working in inpatient wards had significantly higher odds of physical workplace violence than those working in outpatient departments (OR = 2.422, 95% CI: 1.282–4.573, *p* = 0.006). A higher frequency of monthly night shifts was associated with increased odds of physical workplace violence (OR = 1.164, 95% CI: 1.081–1.254, *p* < 0.001), whereas higher perceived organizational support was associated with lower odds of physical workplace violence (OR = 0.968, 95% CI: 0.953–0.983, *p* < 0.001). Detailed results are presented in Table 4.

### 3.7 Univariate analysis of non-physical workplace violence among psychiatric nurses

Univariate analyses showed significant differences in non-physical workplace violence according to sex, marital status, years of work experience, professional title, department, number of monthly night shifts, perceived stress (CPSS), perceived organizational support (SPOS), and positive coping scores (all *p* < 0.05). No significant differences were observed for age, educational level, hospital level, or negative coping scores (all *p* > 0.05). Details are presented in Supplementary Table S3.

### 3.8 Multivariable logistic regression analysis of non-physical workplace violence among psychiatric nurses

Using exposure to non-physical workplace violence (yes/no) as the dependent variable, a multivariable logistic regression model was constructed after appropriate coding of all prespecified covariates (Supplementary Table S1). The results showed that sex, years of work experience, hospital level, department, number of monthly night shifts, perceived organizational support, and negative coping were independently associated with non-physical workplace violence among psychiatric nurses (Table 5).

**Table 5:** Multivariable logistic regression analysis of factors associated with non-physical workplace violence among psychiatric nurses.

Factor	B	SE	Wald *χ*^2^	*p*	OR	95% CI
Sex (reference: female)	0.772	0.251	9.454	0.002	2.165	1.323 ~ 3.543
Age	0.014	0.025	0.322	0.570	1.015	0.965 ~ 1.066
Marital status (reference: divorced)			0.319	0.852		
Unmarried	0.007	0.635	0.000	0.991	1.007	0.290 ~ 3.496
Married	0.134	0.593	0.051	0.821	1.144	0.358 ~ 3.657
Years of work experience (reference: <5 years)			14.679	0.002		
5–10 years	0.681	0.325	4.398	0.036	1.976	1.046 ~ 3.733
11–15 years	1.127	0.416	7.336	0.007	3.085	1.365 ~ 6.971
>15 years	0.388	0.529	0.537	0.464	1.473	0.523 ~ 4.155
Educational level (reference: bachelor’s degree or above)			2.435	0.296		
Secondary specialized school	0.941	0.826	1.296	0.255	2.562	0.507 ~ 12.937
Junior college	0.316	0.266	1.414	0.234	1.372	0.815 ~ 2.309
Professional title(reference: senior)			2.241	0.326		
Junior	−0.561	0.397	2.003	0.157	0.570	0.262 ~ 1.241
Intermediate	−0.263	0.315	0.700	0.403	0.769	0.415 ~ 1.424
Hospital level (reference: tertiary grade A)			10.039	0.018		
Primary	0.246	0.889	0.076	0.782	1.278	0.224 ~ 7.303
Secondary	−0.403	0.211	3.671	0.055	0.668	0.442 ~ 1.009
Tertiary	−0.594	0.201	8.704	0.003	0.552	0.372 ~ 0.819
Department(reference: outpatient)	0.631	0.273	5.342	0.021	1.879	1.101 ~ 3.208
Night shifts (days per month)	0.111	0.035	9.980	0.002	1.118	1.043 ~ 1.198
SPOS score	−0.035	0.008	20.719	<0.001	0.966	0.951 ~ 0.980
CPSS score	0.009	0.012	0.559	0.455	1.009	0.986 ~ 1.033
Positive coping score	−0.026	0.014	3.346	0.067	0.974	0.947 ~ 1.002
Negative coping score	0.041	0.018	5.032	0.025	1.042	1.005 ~ 1.080
Constant	−0.510	1.396	0.134	0.715	0.600	

CI, confidence interval; CPSS, perceived stress score; OR, odds ratio; SPOS, perceived organizational support score.

Specifically, male nurses had significantly higher odds of experiencing non-physical workplace violence than female nurses (OR = 2.165, 95% CI: 1.323–3.543, *p* = 0.002). Compared with nurses with less than 5 years of work experience, those with 5–10 years (OR = 1.976, 95% CI: 1.046–3.733, *p* = 0.036) and 11–15 years of work experience (OR = 3.085, 95% CI: 1.365–6.971, *p* = 0.007) had significantly higher odds of non-physical workplace violence. Compared with nurses working in tertiary grade A psychiatric hospitals, those working in tertiary psychiatric hospitals had significantly lower odds of non-physical workplace violence (OR = 0.552, 95% CI: 0.372–0.819, *p* = 0.003). In addition, nurses working in inpatient wards had significantly higher odds of non-physical workplace violence than those working in outpatient departments (OR = 1.879, 95% CI: 1.101–3.208, *p* = 0.021). A higher frequency of monthly night shifts was associated with increased odds of non-physical workplace violence (OR = 1.118, 95% CI: 1.043–1.198, *p* = 0.002), whereas higher perceived organizational support was associated with lower odds of non-physical workplace violence (OR = 0.966, 95% CI: 0.951–0.980, *p* < 0.001). Higher negative coping scores were also independently associated with greater odds of non-physical workplace violence (OR = 1.042, 95% CI: 1.005–1.080, *p* = 0.025). Detailed results are presented in Table 5.

**Figure 1:**
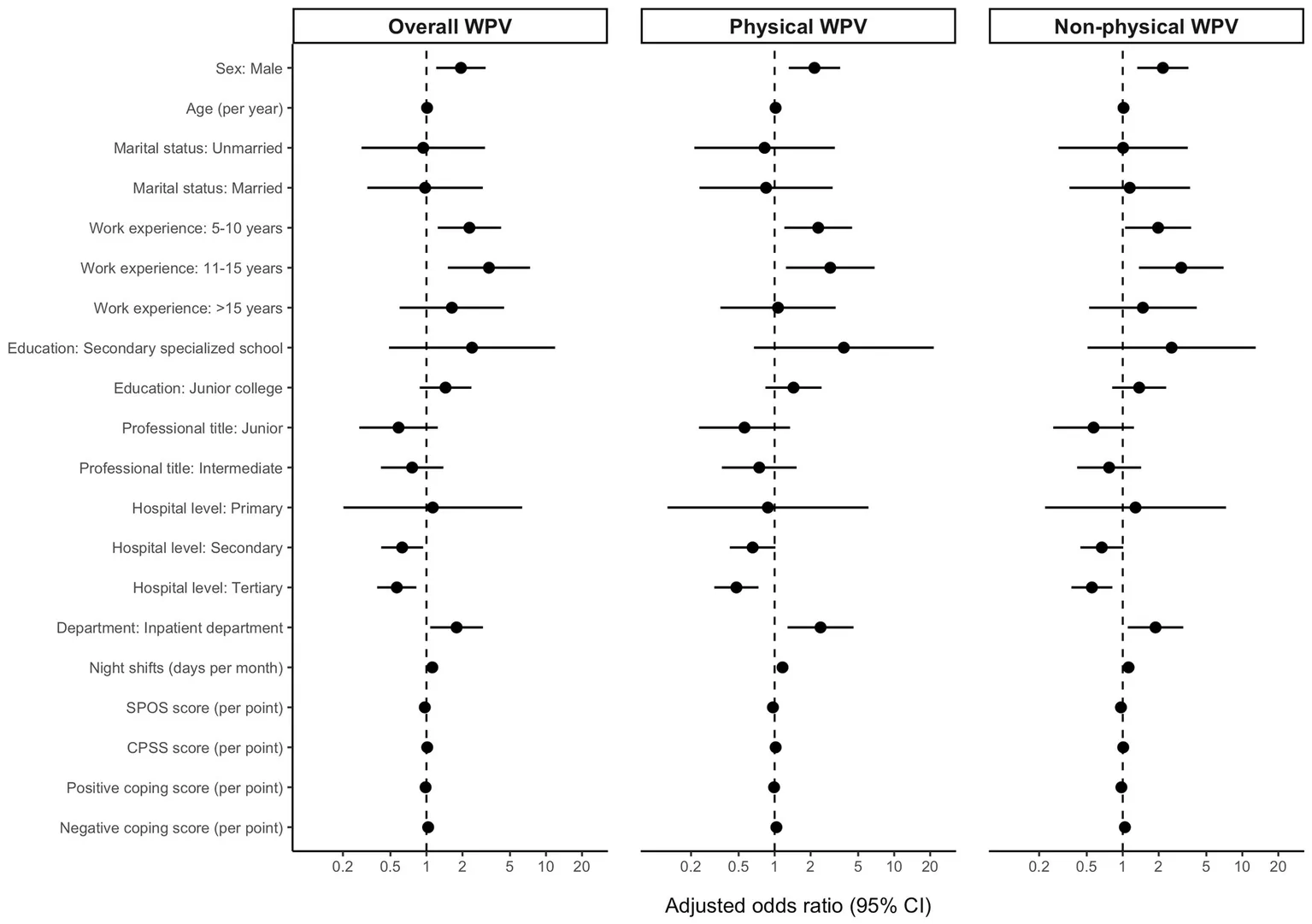
Forest plots of factors associated with overall, physical, and non-physical workplace violence among psychiatric nurses. Points represent adjusted odds ratios, and horizontal lines represent 95% confidence intervals obtained from the multivariable logistic regression models. The dashed vertical line indicates an odds ratio of 1. Reference categories are presented in Tables 3–5.

Across the three multivariable models, negative coping was significantly associated only with non-physical workplace violence, whereas its associations with overall and physical workplace violence were not statistically significant. Positive coping was not significantly associated with any of the three workplace violence outcomes after adjustment for the other covariates. The adjusted odds ratios and 95% confidence intervals from the three multivariable logistic regression models are visually summarized in Figure 1.

### 3.9 Bootstrap sensitivity analyses

Bootstrap sensitivity analyses yielded results consistent with the primary multivariable logistic regression analyses. Male sex remained significantly associated with overall workplace violence (OR = 1.942, BCa 95% CI: 1.224–3.361, *p* = 0.009), physical workplace violence (OR = 2.154, BCa 95% CI: 1.223–4.112, *p* = 0.004), and non-physical workplace violence (OR = 2.165, BCa 95% CI: 1.296–3.955, *p* = 0.001). These findings supported the robustness of the associations despite the unequal sex distribution.

## 4 Discussion

### 4.1 Prevalence of workplace violence among psychiatric nurses

Based on a multicenter sample of nurses from 20 psychiatric hospitals in Jiangsu Province, China, this study systematically examined the prevalence of workplace violence experienced by psychiatric nurses during the past 12 months. The results showed that 43.9% of psychiatric nurses reported experiencing at least one form of workplace violence, indicating that workplace violence remains common among psychiatric nurses.

Previous studies have reported wide variability in the prevalence of workplace violence among psychiatric nurses, ranging from 11.4% to 97.6%. Such variation may be attributable to differences in study populations, definitions of workplace violence, measurement instruments, and reference time frames (35). The prevalence observed in the present study falls within this reported range. Overall, our findings are largely consistent with previous domestic and international studies conducted among healthcare workers and nursing populations, further confirming the widespread and serious nature of workplace violence in psychiatric nursing practice (36).

In terms of violence types, verbal abuse and verbal threats were the most common, while physical assault and sexual harassment were also reported by a considerable proportion of nurses. Notably, some nurses experienced repeated violent incidents within the past year, suggesting that workplace violence among psychiatric nurses is not only prevalent but also recurrent. This phenomenon may be related to the characteristics of psychiatric nursing, including frequent fluctuations in patients’ psychiatric symptoms, an increased likelihood of aggressive behaviors, and prolonged exposure of nurses to high-pressure clinical environments (37).

### 4.2 Influencing factors of workplace violence among psychiatric nurses

In the univariate analyses, sex, years of work experience, professional title, department, number of monthly night shifts, perceived stress, perceived organizational support, and coping styles were significantly associated with workplace violence. After adjustment for all prespecified covariates in the multivariable logistic regression model, sex, years of work experience, hospital level, department, number of monthly night shifts, and perceived organizational support remained independently associated with workplace violence. These findings suggest that workplace violence among psychiatric nurses is associated with multiple individual and work-related factors.

Sex was independently associated with workplace violence, with male nurses having higher odds of experiencing workplace violence than female nurses (OR = 1.942). This finding is consistent with several previous studies conducted in both domestic and international contexts (38, 39). Rather than indicating an inherent effect of sex, this association may partly reflect a gendered division of labor within psychiatric nursing. Male nurses may be more frequently involved in tasks such as implementing protective restraints, caring for agitated or impulsive patients, managing conflicts, supervising high-risk patients, and responding to emergencies, which may increase their direct exposure to potentially violent situations (40). Previous research among psychiatric nurses also found that male nurses were more likely to respond to aggressive patients and experience physical and verbal assaults, whereas female nurses spent more time building rapport with patients and developing cooperative relationships with colleagues (41). Such differences in task allocation and occupational expectations may partly explain why sex remained independently associated with all three types of workplace violence. However, the present study did not directly assess specific task assignments, involvement in restrictive interventions, patient acuity, or contact with aggressive patients. Therefore, this explanation should be regarded as plausible rather than as a confirmed mechanism.

Work experience was also independently associated with workplace violence. Compared with nurses with less than 5 years of work experience, those with 5–10 years (OR = 2.287) and 11–15 years (OR = 3.330) of work experience had significantly higher odds of workplace violence. This pattern may reflect differences in professional roles across career stages. In clinical practice, nurses with 5–15 years of experience often serve as core frontline staff and assume a greater proportion of high-risk patient care tasks, conflict management responsibilities, and night shifts, resulting in increased exposure to violent incidents. In contrast, nurses with more than 15 years of work experience are more likely to transition into managerial, teaching, or coordination roles, with reduced involvement in high-risk procedures and direct patient contact, thereby reducing their direct exposure to potentially violent situations. Moreover, cumulative occupational fatigue and psychological strain associated with prolonged high-intensity work may further increase vulnerability to violent encounters during this career stage (42). In addition, compared with nurses working in outpatient departments, those working in inpatient wards had higher odds of experiencing workplace violence (OR = 1.786). A higher frequency of monthly night shifts was also independently associated with workplace violence (OR = 1.119). These findings may be explained by the higher intensity of direct patient contact, greater symptom instability among psychiatric inpatients, relatively insufficient staffing during night shifts, and limited availability of resources for managing emergencies, all of which may increase the likelihood of violent incidents in psychiatric settings (43).

Hospital level was also independently associated with workplace violence. Compared with nurses working in tertiary grade A psychiatric hospitals, those working in tertiary psychiatric hospitals had lower odds of experiencing workplace violence (OR = 0.565). One possible explanation is that differences in patient case-mix, staffing levels, security measures, and organizational management may exist across hospital levels, which could influence nurses’ exposure to workplace violence. However, the underlying mechanisms remain unclear, and further studies are warranted to explore the potential institutional factors contributing to these differences (44).

Perceived organizational support was independently associated with lower odds of overall, physical, and non-physical workplace violence. When nurses perceive stronger organizational support, their needs for respect, autonomy, and a sense of belonging are more likely to be met (45). This association may extend beyond nurses’ general satisfaction with their organizations. Visible organizational support may increase nurses’ willingness to report early warning signs, seek assistance, communicate risks to colleagues, and adopt team-based responses before an escalating situation develops into violence. It may also strengthen nurses’ confidence that violent incidents will be investigated fairly and that affected staff will receive assistance rather than blame. Previous qualitative evidence suggests that formal organizational support following violent incidents may sometimes be delayed, inadequate, or insufficiently visible to nurses (46).

From a practical perspective, psychiatric hospitals could translate organizational support into visible and accessible measures, including adequate staffing and access to experienced personnel during high-risk shifts, regular training in violence risk assessment and de-escalation, rapid-response procedures for escalating incidents, and improved environmental and security measures in high-risk wards. Hospitals should also establish clear, accessible, and non-punitive reporting systems. Following a violent incident, affected nurses should receive timely managerial follow-up, structured debriefing, medical assessment, psychological counselling, and peer support. Managers should provide feedback regarding the actions taken after reported incidents so that nurses can see that reporting leads to meaningful organizational responses. Comprehensive workplace violence prevention should combine staff training with managerial support, hazard prevention, sustained follow-up, and post-incident care rather than relying on individual-level training alone (47). Nevertheless, because of the cross-sectional design, the present study cannot establish that strengthening perceived organizational support would directly prevent workplace violence.

### 4.3 Comparative analysis of influencing factors across different types of workplace violence

Physical and non-physical workplace violence shared several associated factors, including sex, years of work experience, hospital level, department, number of monthly night shifts, and perceived organizational support. This similarity suggests that both forms of violence may arise from overlapping individual, environmental, and organizational conditions. Nevertheless, the different findings regarding coping styles suggest that the pattern of associated factors may vary according to the type of violence.

One notable difference was that negative coping was independently associated only with non-physical workplace violence. Non-physical violence, including verbal abuse, threats, intimidation, and humiliation, may involve repeated interpersonal stressors and sustained emotional demands. Nurses who rely more heavily on negative coping strategies may experience greater difficulty with communication, emotional regulation, and conflict resolution during nurse–patient interactions, which may increase their vulnerability to non-physical workplace violence (48). In contrast, negative coping was not independently associated with physical workplace violence, suggesting that physical assaults may be more strongly influenced by patient-related clinical characteristics and immediate work-environment factors than by individual coping styles (49). However, the observed association may also be bidirectional, because repeated exposure to non-physical violence could reinforce negative coping responses. Therefore, the direction of this association cannot be determined from the present cross-sectional data. Positive coping was not independently associated with any of the three workplace violence outcomes after adjustment for the other covariates, suggesting that individual coping strategies alone may be insufficient to offset adverse workplace and organizational conditions.

Taken together, these findings indicate that physical and non-physical workplace violence share several associated factors but may also show partly different patterns. Prevention strategies should therefore combine system-level measures with interventions targeting specific forms of violence. Regular de-escalation training may help nurses recognize early signs of aggression, establish therapeutic communication, and safely manage potentially violent situations (50). Simulation-based education may further improve nurses’ confidence, preparedness, and ability to manage aggressive behaviors in realistic clinical scenarios (51). For non-physical violence, additional attention should be given to repeated verbal and psychological aggression, communication support, and the identification of negative coping responses. These individual-level strategies should complement, rather than replace, organizational responsibility for providing a safe working environment.

### 4.4 Limitations

This study has several limitations. First, the cross-sectional design precludes causal inference or determination of temporal relationships between workplace violence and the associated factors identified in this study. Therefore, the observed associations should be interpreted with caution, and future longitudinal or prospective studies are needed to establish temporality and confirm these findings. Second, all variables were collected simultaneously through a single self-administered online survey. Although validated instruments and standardized survey procedures were used, the findings may be affected by recall bias, social desirability bias, and common method variance. Harman’s single-factor test did not indicate substantial common method variance; however, this statistical test cannot completely exclude such bias. Third, although this was a multicenter study involving 20 psychiatric hospitals, all participants were recruited from Jiangsu Province, China, and tertiary grade A psychiatric hospitals accounted for a relatively large proportion of the sample. Consequently, the findings may not be fully representative of psychiatric nurses across China, and their generalizability to other regions or healthcare settings should be interpreted with caution. Future nationwide multicenter studies with more geographically diverse and representative samples are warranted. Fourth, although we adjusted for a broad range of individual and work-related characteristics, several potentially important ward- and institutional-level factors, including the patient-to-nurse ratio, patient case-mix, ward management practices, security measures, and institutional workplace violence prevention policies, were not collected and therefore could not be included in the analyses. These unmeasured organizational characteristics may have confounded the observed associations, particularly those related to department type and physical workplace violence, and residual confounding cannot be excluded. Fifth, the number of male nurses was substantially smaller than that of female nurses. This imbalance may have reduced the precision of the sex-related estimates and increased their sensitivity to sampling variability. Although bootstrap sensitivity analyses produced estimates consistent with the primary analyses, bootstrap resampling cannot eliminate the underlying imbalance in the observed sample. Therefore, the findings regarding sex should be interpreted cautiously and confirmed in future studies with larger and more balanced samples of male and female psychiatric nurses.

Despite these limitations, this study provides evidence from a large multicenter sample of psychiatric nurses and offers important insights into the prevalence of and factors associated with workplace violence. The findings contribute to the existing evidence base and provide a useful foundation for future longitudinal studies and the development of evidence-based prevention and intervention strategies in psychiatric healthcare settings.

## 5 Conclusion

Workplace violence remains common among psychiatric nurses, and its occurrence is associated with a combination of individual, occupational, and organizational factors. Physical and non-physical workplace violence shared several associated factors but may involve partly different underlying mechanisms. Physical violence appeared to be more closely related to work-environmental and immediate patient-care conditions, including inpatient work and frequent night shifts, whereas non-physical violence was additionally associated with negative coping. Perceived organizational support was consistently associated with lower odds of both types of violence. These findings suggest that prevention strategies should combine system-level measures, such as adequate staffing, safer work environments, effective incident-response procedures, and organizational support, with targeted interventions addressing communication, coping responses, and repeated verbal or psychological aggression.

## Data Availability

The original contributions presented in the study are included in the article/Supplementary material, further inquiries can be directed to the corresponding authors.
