# Supplementary material for: Prevalence and influencing factors of workplace violence among psychiatric nurses in China: a cross-sectional study
Source: Front Public Health. 2026 Aug 12;14:1822574. doi: 10.3389/fpubh.2026.1822574 (PMC13506702; doi:10.3389/fpubh.2026.1822574)
Supplement: Supplementary file 1 [file Table_1.DOCX]

Supplementary Material

# 1 Supplementary Tables

**Supplementary Table S1. Variable coding for multivariate logistic regression analyses**

| Variable | Coding |
| --- | --- |
| Sex | 1 = Male, 2 = Female |
| Age | Entered as continuous variable |
| Marital status | 1=Unmarried, 2=Married, 3=Divorced |
| Years of work experience | 1 = <5 years, 2 = 5–10 years, 3 = 11–15 years, 4 = >15 years |
| Educational level | 1=Secondary specialized school, 2=Junior college, 3=Bachelor's degree or above |
| Professional title | 1 = Junior, 2 = Intermediate, 3 = Senior |
| Hospital level | 1=Primary, 2=Secondary, 3=Tertiary, 4=Tertiary grade A |
| Department | 1 = Outpatient department, 2 = Inpatient ward |
| Number of night shifts (days) | Entered as continuous variable |
| Perceived stress score (CPSS) | Entered as continuous variable |
| Perceived organizational support score (SPOS) | Entered as continuous variable |
| Positive coping score | Entered as continuous variable |
| Negative coping score | Entered as continuous variable |

**Supplementary Table S2. Univariate analysis of factors associated with physical workplace violence among psychiatric nurses during the past 12 months (n = 749)**

| Variable | Categorization | Physical violence group (n = 279) | Non-violence group  group (n = 470) | χ^2^/t | P |
| --- | --- | --- | --- | --- | --- |
| Sex | Male | 53 | 42 | 15.999 | <0.001 |
|  | Female | 226 | 428 |  |  |
| Age (years) |  | 33.23±6.84 | 33.60±8.18 | 0.664 | 0.507 |
| Marital status | Unmarried | 74 | 141 | 1.378 | 0.502 |
|  | Married | 199 | 322 |  |  |
|  | Divorced | 6 | 7 |  |  |
| Years of work experience | < 5 years | 36 | 102 | 28.449 | <0.001 |
|  | 5–10 years | 104 | 144 |  |  |
|  | 11–15 years | 79 | 76 |  |  |
|  | > 15 years | 60 | 148 |  |  |
| Educational level | Secondary specialized school | 3 | 4 | 1.369 | 0.498 |
|  | Junior college | 43 | 88 |  |  |
|  | Bachelor's degree or above | 233 | 378 |  |  |
| Professional title | Junior | 139 | 249 | 2.876 | 0.237 |
|  | Intermediate | 120 | 176 |  |  |
|  | Senior | 20 | 45 |  |  |
| Hospital level | Primary hospital | 2 | 3 | 4.173 | 0.243 |
|  | Secondary hospital | 94 | 94 |  |  |
|  | Tertiary hospital | 116 | 116 |  |  |
|  | Tertiary grade A hospital | 257 | 257 |  |  |
| Night shifts (days) |  | 4.83±2.44 | 3.75±2.66 | -5.648 | <0.001 |
| Department | Outpatient department | 15 | 60 | 10.610 | 0.001 |
|  | Inpatient ward | 264 | 410 |  |  |
| CPSS score | / | 39.17±6.97 | 36.75±8.12 | -4.313 | <0.001 |
| SPOS score | / | 48.04±12.84 | 54.25±13.41 | 6.216 | <0.001 |
| Positive coping score | / | 34.28±7.87 | 37.17±8.06 | 4.772 | <0.001 |
| Negative coping score | / | 19.38±4.92 | 18.94±5.48 | -1.089 | 0.277 |

Abbreviation: CPSS: Perceived stress score; SPOS: Perceived organizational support score

**Supplementary Table S3. Univariate analysis of factors associated with non-physical workplace violence among psychiatric nurses during the past 12 months (n = 802)**

| Project | Categorization | Non-physical violence group(n=332) | | Non-violence group  (*n*=470) | χ^2^/t | P |
| --- | --- | --- | --- | --- | --- | --- |
| Sex | Male | 56 | 42 | | 11.410 | 0.001 |
|  | Female | 276 | 428 | |  |  |
| Age (years) |  | 34.34±7.23 | 33.60±8.18 | | -1.364 | 0.173 |
| Marital status | Unmarried | 73 | 141 | | 6.931 | 0.031 |
|  | Married | 251 | 322 | |  |  |
|  | Divorced | 8 | 7 | |  |  |
| Years of work experience | < 5 years | 37 | 102 | | 30.082 | <0.001 |
|  | 5–10 years | 104 | 144 | |  |  |
|  | 11–15 years | 99 | 76 | |  |  |
|  | > 15 years | 92 | 148 | |  |  |
| Educational level | Secondary specialized school | 4 | 4 | | 3.926 | 0.140 |
|  | Junior college | 45 | 88 | |  |  |
|  | Bachelor's degree or above | 283 | 378 | |  |  |
| Professional title | Junior | 145 | 249 | | 8.028 | 0.018 |
|  | Intermediate | 157 | 176 | |  |  |
|  | Senior | 30 | 45 | |  |  |
| Hospital level | Primary hospital | 3 | 3 | | 2.624 | 0.453 |
|  | Secondary hospital | 75 | 94 | |  |  |
|  | Tertiary hospital | 67 | 116 | |  |  |
|  | Tertiary grade A hospital | 187 | 257 | |  |  |
| Night shifts (days) |  | 4.49±2.58 | 3.75±2.66 | | -3.906 | <0.001 |
| Department | Outpatient | 26 | 60(29.8) | | 4.949 | 0.026 |
|  | Inpatient ward | 306 | 410(70.2) | |  |  |
| CPSS score | / | 39.17±6.97 | 36.75±8.12 | | -4.197 | <0.001 |
| SPOS score | / | 47.18±12.65 | 54.25±13.41 | | 7.514 | <0.001 |
| Positive coping score | / | 33.74±7.46 | 37.17±8.06 | | 6.104 | <0.001 |
| Negative coping score | / | 19.18±4.69 | 18.94±5.48 | | -0.642 | 0.521 |

Abbreviation: CPSS: Perceived stress score; SPOS: Perceived organizational support score
